# Effectiveness of a 5G Local Area Network–Based Digital Microscopy Interactive System: Quasi-Experimental Design

**DOI:** 10.2196/70256

**Published:** 2025-12-24

**Authors:** Jie Xu, Jihong Sha, Song Jia, Jiao Li, Lei Xu, Zhihua Shao

**Affiliations:** 1 Teaching Laboratory Center School of Medicine Tongji University Shanghai China; 2 Department of Cell Biology School of Medicine Tongji University Shanghai China

**Keywords:** microscopy technique, digital interactive system, 5G, medical education, undergraduate

## Abstract

**Background:**

Technological innovation is reshaping the landscape of medical education, bringing revolutionary changes to traditional teaching methods. In this context, the upgrade of the teaching model for microscopy, as one of the core skills in medical education, is particularly important. Proficiency in microscope operation not only affects medical students’ pathology diagnosis abilities but also directly impacts the precision of surgical procedures and laboratory analysis skills. However, current microscopy pedagogy faces dual challenges: on one hand, traditional teaching lacks real-time image sharing capabilities, severely limiting the effectiveness of immediate instructor guidance; on the other hand, students find it difficult to independently identify technical flaws in their operations, leading to inefficient skill acquisition. Although whole-slide imaging-based microscopy system technology has partially addressed the issue of image visualization, it cannot replicate the tactile feedback and physical interaction experience of the real world. The breakthrough development of 5G communication technology—with its ultrahigh transmission speed and ultralow latency—provides an innovative solution to this teaching challenge. Leveraging this technological advantage, Tongji University’s biology laboratory has pioneered the deployment of a 5G local area network (LAN)–supported digital interactive microscopy system, creating a new model for microscopy education.

**Objective:**

This study aims to investigate the efficacy of an innovative 5G LAN-powered interactive digital microscopy system in enhancing microscopy training efficiency, evaluated through medical students’ academic performance and learning experience.

**Methods:**

Using a quasi-experimental design, we quantify system effectiveness via academic performance metrics and learning experience dimensions. A total of 39 students enrolled in the biology course were randomly assigned to 2 groups: one using traditional optical microscopes (control) and the other using the digital microscopy interactive system (DMIS). Their academic performance was evaluated through a knowledge test and 3 laboratory reports. A 5-point Likert-scale questionnaire was used to gather feedback on students’ learning experiences. In addition, the DMIS group was required to evaluate the specific functions of the system.

**Results:**

In the knowledge test, no statistical difference was found between the 2 groups; however, the DMIS group scored significantly higher in Lecture 2 (*P*<.05). In the laboratory reports, the DMIS group performed significantly better than the control group (mean 90.33, SD 2.63 vs mean 80.53, SD 3.52, *P*<.001). Questionnaire results indicated that the DMIS group has a positive evaluation of the system and expressed greater confidence in its future application. For the evaluation of the laboratory lectures, the DMIS group received higher evaluations on the course content and self-efficacy (*P*<.05), and higher satisfaction with the laboratory lectures (*P*<.05).

**Conclusions:**

Overall, the digital microscope interactive system enhances students’ learning experiences and improves their academic performance. It offers various interactive functions to facilitate the organization of teaching activities and promote immediate feedback in the classroom. Thus, it is a promising tool for microscopy laboratory teaching.

## Introduction

Emerging technologies are rapidly proliferating, elevating the quality of medical training and better preparing students for future clinical practice [1]. For instance, whole-slide imaging-based microscopy system is accepted as a major technique in the teaching of pathology, anatomy, and histology in several countries [2]. A multimedia-supported manikin system capable of simulating a real clinical operating environment was reported to help students reduce barriers to entering the clinic [3]. Furthermore, 3D printing technology, in addition to its application in clinical practice, provides educators with a cost-effective and efficient educational tool [4]. The maturity of multiple technologies has enabled the potential for technical upgrades to microscopy as a fundamental teaching tool.

Microscopy provides a methodological foundation for high-resolution observation and quantitative analysis of microscopic phenomena [5]. As a core competency for medical students, this technique enhances skill acquisition in pathological diagnostics through microstructural visualization, improves intraoperative spatial localization precision via microscope operational adjustments, and supplies essential analytical capabilities for indispensable scientific experimentation. However, current microscopy instruction faces significant challenges: traditional optical training suffers from delayed feedback, as instructors cannot monitor student operations in real time. This leads to undetected technical errors until learners proactively seek guidance [6]. Compounding this issue, beginners lack the expertise to self-identify technical deficiencies [7], creating a dual problem of instructor feedback absence and limited student self-assessment. Such gaps risk entrenching structural misconceptions. While whole-slide imaging-based microscopy system enables image visualization, its inherent limitations remain critical: it fails to replicate optical parameter adjustment processes and lacks tangible tactile feedback [8], thereby undermining practical skill development. Microscopy expertise necessitates not only the physical ability to visualize specimens but also the cognitive capacity to explore and discriminate among potential morphological variations for accurate characterization of target samples. This dual requirement implies that both knowledge internalization and operational proficiency constitute core learning objectives in microscopy instruction, whereas current pedagogical approaches demonstrate limitations in concurrently achieving these competencies.

The digital microscope interactive system, based on optical microscopes, is a multiterminal digital imaging device connected by 5G local area network (LAN). The system retains the tactile sensation of physical operation while enabling the display of microscopic images on software-equipped devices (such as computers, tablets, or mobile phones) through analog-to-digital conversion. Leveraging 5G’s high-bandwidth connectivity, the system enables instantaneous image transmission between terminals. Consequently, instructors can simultaneously observe all students’ microscope images on the teacher-terminal computer. This real-time monitoring capability enables immediate detection of operational errors, even in the absence of student feedback. Beyond real-time oversight, the multiplatform compatibility supports interactive pedagogical functions such as distributing laboratory instructions, uploading resulting images, demonstrating operations, and so on. The high popularity of smartphones among students currently makes this mode both flexible and economical [9].

Previous reports have documented the application of 5G technology in multiple educational scenarios [10,11]. While a digital microscope system has been implemented in the pathogenic biology laboratory in China [12], no research has yet reported the deployment of 5G LAN-based digital interactive microscopy systems in laboratory teaching. Consequently, investigating 5G-enabled interactive microscopy systems embodies significant innovative potential to enhance instructional efficacy by addressing the aforementioned limitations in microscopy instruction.

Microscopy instruction in medical education faces challenges of delayed instructor feedback and limited student self-correction capabilities, which impact skill acquisition efficiency. To address this challenge, Tongji University innovatively deployed a 5G LAN-based digital microscopy interaction system (DMIS). This system preserves the tactile sensation of physical operation while leveraging digital microscopy for procedural visualization. Capitalizing on 5G’s high-speed transmission, it establishes an immediate feedback mechanism, thereby enhancing instructional efficiency in microscopy training. Using a quasi-experimental design—frequently applied in education [13,14]—this study compared the instructional efficacy between this digital system and conventional optical microscopy training. We hypothesize that these technological features will significantly improve pedagogical outcomes. A comparative analysis of academic performance and learning experience questionnaire data between the DMIS group and the traditional training control group provides evidence-based guidance for medical educators reliant on microscopy technology to optimize pedagogical approaches.

## Methods

### Experimental Procedures and Participants

The biology course for medical undergraduates at Tongji University is a second-year component of the curriculum, comprising 10 theoretical sessions and 3 laboratory sessions. These laboratory sessions are aligned with the theoretical learning schedule and consist of 4 periods for each session, including the use of microscopes and cell cycle analysis, cell counting and cell culture, and mouse chromosome preparation and karyotype analysis (see Table 1). Students are organized into classes of 20, with 2 classes running simultaneously. In each class, the instructor introduces the experimental content and principles, followed by a demonstration of the procedure. Students then work in pairs to conduct the experiments and complete their laboratory reports according to the requirements outlined in the Microsoft PowerPoint slides provided by the instructor. All activities described in this article have received informed consent from the students.

**Table 1:** Laboratory experiments.

Experiment name	Experimental activities
The use of a microscope and cell cycle analysis.	Microscopy training with commercially prepared slides. Capture clear and accurate images of each stage of mitosis.
Cell counting and cell culture.	Prepare the cell sample and count viable cells using microscopy. Cell passaging.
Preparation and observation of mouse chromosomes.	Prepare and stain mouse chromosomes. Capture clear images of mouse chromosomes.

### Digital Microscopy Interactive System

**Figure 1:**
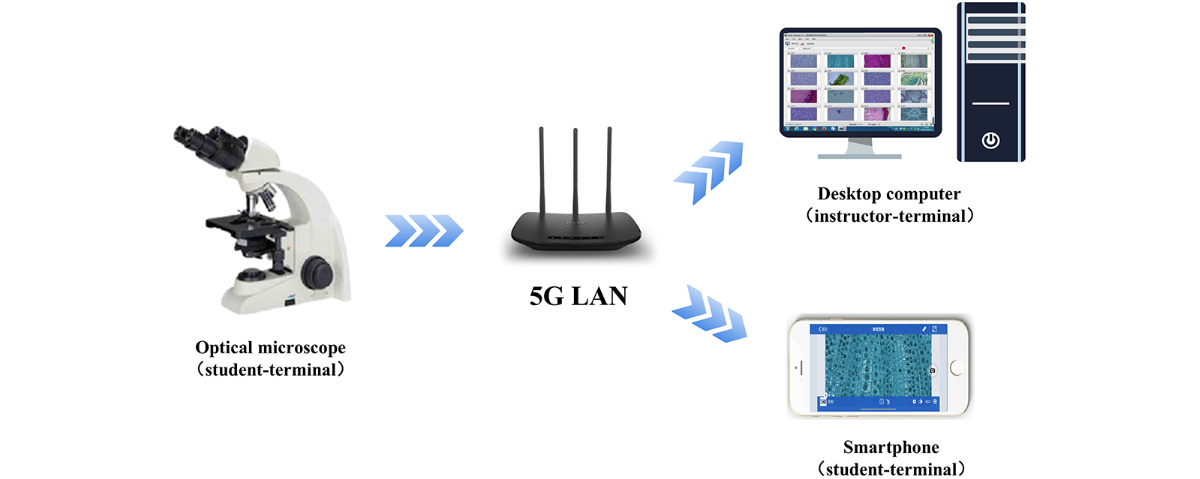
The framework of a digital microscopy interactive system based on 5G local area network (LAN).

**Figure 2:**
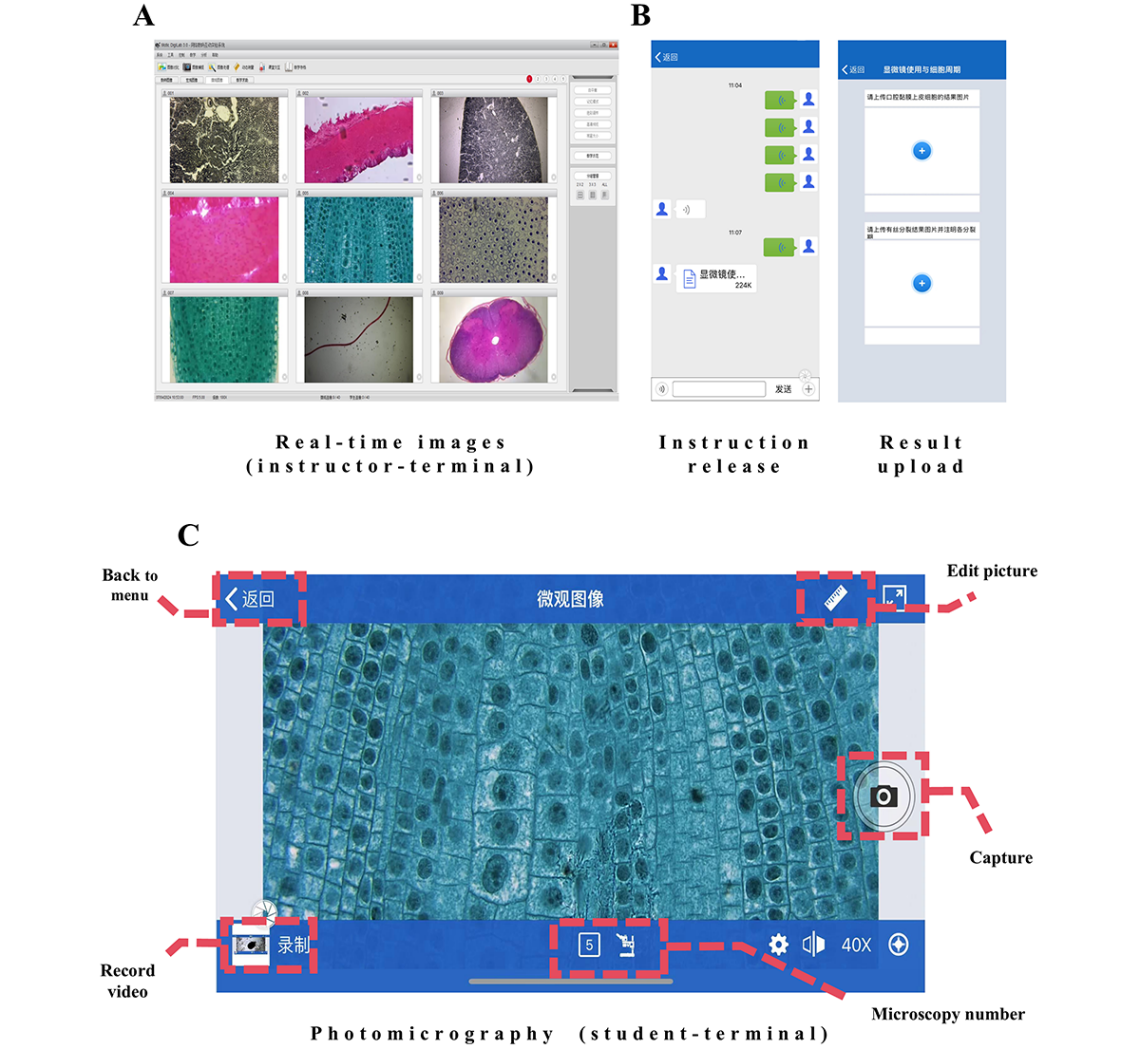
The function of digital microscopy interactive system.

The digital microscopy interaction system primarily consists of 3 parts: Panthera I series optical microscopes (Motic Corp), Motic Digilab 3.0 software (Motic Corp), and a computer or mobile device, all of which can connect via a 5G LAN (see Figure 1). In class, students need to download and install the software from the app store onto their digital devices (eg, smartphones and tablets), connect to the designated 5G LAN, and enter their microscope number in the software to view live microscopy images. This setup enables real-time sharing of all microscopic images across the network. Simultaneously, the instructor's terminal computer with the installed software can display all microscope images currently in use, allowing both students and the instructor to visualize the live microscopic manipulations at the same time (see Figure 2A).

The Motic Digilab 3.0 software supports 4 functions for both instructors and students (see Table 2). On the student side, students can capture live images, record videos, and ask questions within the interface (see Figure 2C). On the instructor side, instructors can monitor the progress of all students and address issues based on their live images. Since both sides are connected to the same 5G LAN, they can transfer files to each other, thereby enhancing the efficiency of teaching activities (see Figure 2B).

**Table 2:** The functions of the digital microscopy interactive system.

Function	Instructors’ terminal	Students’ terminal
Microimage	View images observed by all student-terminal microscopes in real time.	View live images observed under the microscope on its mobile app.
Macroimage	View videos recorded by all student-terminal mobile phones.	Record the experimental process with a mobile phone camera.
Demonstration	Use the instructor-terminal microscope to demonstrate proper operation in either forced or unforced mode.	Continue to use the microscope, while students who have difficulties can refer to the instructor’s demonstration.
Interaction	Deliver the protocol and requirements of this experiment. Instruct students through their real-time microscope images.	Upload the result images of this experiment as required. Ask questions by text or voice in their mobile app.

### Grouping

The biology course, conducted in the second semester of the 2023-2024 academic year, involved a total of 39 medical freshmen. In this study, randomization was performed using a computer-generated randomization program integrated within the school education management system. Concealment was ensured by implementing the allocation through the school’s system without any manual intervention or researcher influence. Participants were randomly assigned to 2 groups: the DMIS group, which used the digital microscope interactive system, and the control group, which used the traditional optical microscope.

### Evaluation

The assessment framework (see Table 3) for the DMIS, designed around the instructional objectives of mastering operation and internalizing knowledge, comprises 4 key components. Specifically, student academic performance was jointly assessed using 1 knowledge test and 3 laboratory reports used to evaluate the internalization of knowledge and proficiency in operation. Furthermore, student learning experience feedback is collected via a 5-point Likert scale questionnaire. In addition, students in the DMIS group complete a supplementary 5-point Likert scale questionnaire specifically designed to evaluate the system’s functional features.

**Table 3:** The evaluation framework of the digital microscopy interactive system.

Dimension	Testing method	Participants	Measure analyzed
Knowledge	Knowledge test	All students	Comprehension of knowledge and mastery of operational protocols.
Skill	Laboratory reports	All students	Microscope operation proficiency and accuracy in result analysis.
Attitude	A 5-point Likert scale questionnaire	All students	Course satisfaction.
Functional evaluation	A 5-point Likert scale questionnaire	DMIS^a^ group	System capabilities and future applications.

^a^DMIS: digital microscopy interactive system.

The knowledge test was jointly designed by all instructors participating in the Biology course instruction. To assess comprehension of structural knowledge and mastery of operational protocols, 6 questions were implemented per laboratory lecture. It has been reported that multiple true-false questions are superior in revealing students' misunderstanding of knowledge [15,16]. Therefore, a total of 18 true-false questions were designed. After class, each student anonymously completed the online test through the Sojump platform at the same time.

Their laboratory reports were submitted to the Canvas learning management system at Tongji University, enabling all course instructors to access and evaluate these documents online. The assessment rubric, collaboratively developed by the faculty members, primarily emphasizes microscope operation proficiency and accuracy in result analysis. Each dimension was assessed using a 4-tier performance scale: excellent (45 points), proficient (40 points), satisfactory (35 points), and unsatisfactory (30 points). This contributed to a maximum of 50 points per dimension, thereby yielding a total possible score of 100 points. Student competence in microscopy techniques was evaluated through the scoring of micrographs presented in the reports. High-quality microscopic images reflect proper adjustment of the microscope to achieve optimal resolution, appropriate magnification, and adequate illumination during image capture. Interpretation of results demonstrates students’ understanding of target cell biological characteristics and their proficiency in identifying and locating cellular structures through microscopic techniques.

All participants anonymously completed a 5-point Likert scale questionnaire assessing their learning experiences in laboratory lectures. It encompassed four domains: (1) course content, (2) teaching quality, (3) self-efficacy, and (4) teaching effectiveness, with 3-4 items per domain. Specifically, course content assessed the difficulty level, scheduling, interest, and challenge of the laboratory lectures; teaching quality evaluated classroom organization, instructional clarity, and comprehensibility; self-efficacy measured students’ confidence in completing tasks and resolving challenges; and teaching effectiveness gauged whether students had a better understanding of knowledge, techniques, and laboratory safety. Finally, an open-ended item invited students to share which technology they have benefited the most from in their classroom learning.

Functional evaluation of the digital microscopy interactive system was conducted by the DMIS group via a 5-point Likert scale questionnaire. This anonymously collected questionnaire assessed user perceptions of system capabilities and future applications.

### Analysis

The Sojump platform automatically scored students’ responses against predetermined answers and converted results to percentage scores. The Canvas learning management system recorded laboratory report grades based on instructors’ online assessments. Open-ended responses regarding “What technology has benefited you the most in classroom learning?” were analyzed through word frequency analysis provided by the Sojump platform. All data were statistically analyzed using IBM SPSS Statistics (version 26). Group comparisons were performed using the independent samples *t* test for normally distributed continuous variables, while the nonparametric test, such as the Mann-Whitney *U* test, was used for categorical variables or continuous variables deviating from normality. The internal consistency of questionnaire items was evaluated using the Cronbach alpha coefficient (Cronbach α), with Cronbach α≥0.70 considered acceptable for scale reliability. Statistical significance was determined at *P*<.05. Data are presented as mean (SD).

### Ethical Considerations

This study was conducted in accordance with established ethical principles, with participation being voluntary in nature. All participants received comprehensive information regarding the study’s objectives, procedures, and potential implications. They were assured of their right to withdraw at any stage without penalty. The confidentiality of the data was rigorously maintained through the anonymization of all responses, which were subsequently analyzed and reported in aggregated form.

All procedures involving human subjects were carried out in compliance with the ethical standards set forth by relevant national research committees [17]. The study strictly followed applicable ethical guidelines and data protection laws, thereby safeguarding participants’ rights and privacy throughout the research process.

## Results

### Participants’ Demographics

A total of 39 students participated in this course, with 19 in the control group and 20 in the DMIS group. All participants were approximately aged 20 (mean 19.56, SD 0.50) years, with the control group aged 19.74 (SD 0.45) years and the DMIS group aged 19.40 (SD 0.50) years. The demographics of all participants are presented in Table 4. The two groups had a similar composition in terms of gender and GPA (grade point average), which were confirmed to show no statistically significant difference (*P*>.05). Previous academic performance (mean score of previous courses) also showed no significant difference between the groups (*P*>.05), with mean scores of 82.09 (SD 5.49) and 80.69 (SD 5.58) for the control and DMIS groups, respectively.

**Table 4:** The demographics of the participants.

Variable			Total (n=39), n (%)		Control group (n=19), n (%)		DMIS^a^ group (n=20), n (%)		*P* value	
Students			39 (100)		19 (100)		20 (100)		—^b^	
**Sex**										.66^c^
	Male	15 (38.5)		6 (37.5)		7 (35)				
	Female	24 (61.5)		10 (62.5)		13 (65)				
**GPA^d^**										.71^e^
	≧4.0	12 (30.8)		6 (37.5)		5 (25)				
	3.5-3.9	18 (46.2)		7 (43.8)		10 (50)				
	＜3.5	9 (23.1)		3 (18.8)		5 (25)				

^a^DMIS: digital microscopy interactive system.

^b^Not applicable.

^c^Independent sample *t* test.

^d^GPA: grade point average.

^e^Chi-square test.

### Comparison of the Academic Performance Between the Control Group and the DMIS Group

**Figure 3:**
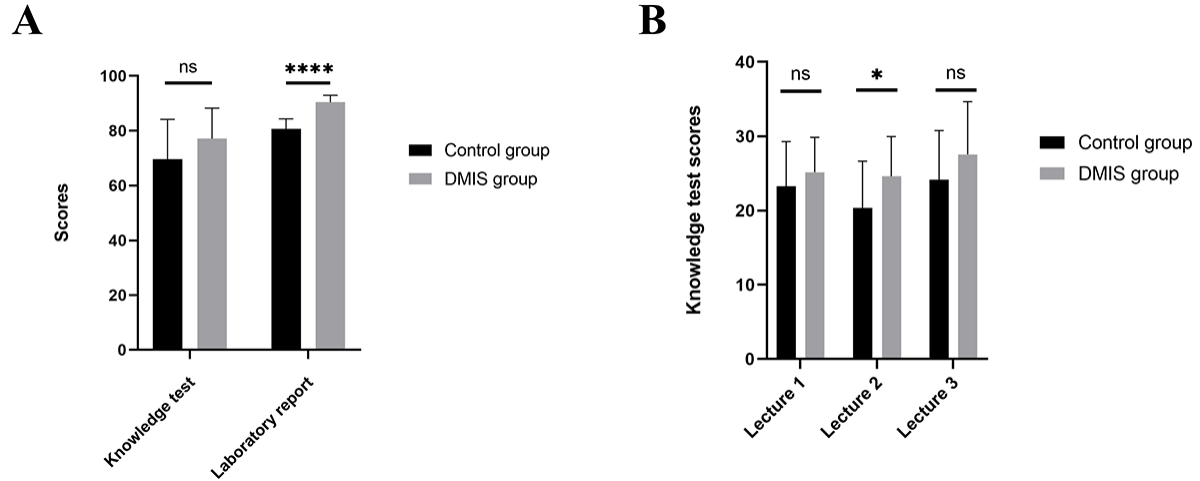
Comparison of scores between the control group and the DMIS group. DMIS: digital microscopy interactive system.

All 39 students took the knowledge test and submitted their laboratory report. In the knowledge test, the DMIS group had a higher mean score of 77.15 (SD 11.10) compared to 69.58 (SD 14.56) for the control group; however, this difference was not statistically significant (*P*=.075; see Figure 3A). To evaluate the system’s potential impact, we assessed group performance across lectures. The DMIS group demonstrated significantly higher mean scores than controls in Lecture 2 (*P*<.05; see Figure 3B), which focused on microscopy-based enumeration of viable cells in prepared samples. For the performance of laboratory reports, the DMIS group scored 90.33 (SD 2.63) versus 80.53 (SD 3.52) for the control group (*P*<.001; see Figure 3A). Overall, the DMIS group achieved better results in both tests and reports.

### Functional Evaluation of This Digital Microscopy Interactive System

**Figure 4:**
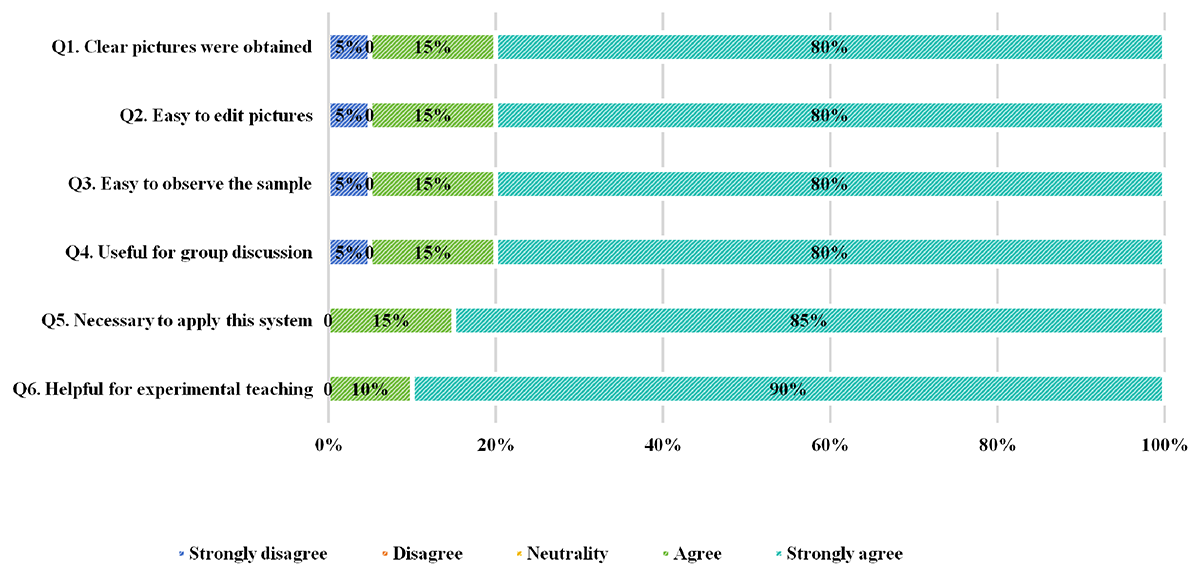
Responses of the digital microscopy interactive system (DMIS) group students regarding the DMIS.

To capture user perspectives regarding the system’s capabilities and future application, we distributed a questionnaire survey to the DMIS group. All participants (100%, 20/20) completed the questionnaire, and its Cronbach α was 0.91, indicating good reliability. In their feedback, most students provided positive evaluations and expressed satisfaction with its functions (see Figure 4 Q1-Q4). They indicated that it contributed positively by capturing clear images, providing easy-to-edit images, facilitating sample observation, and promoting group discussions. (95% agree or strongly agree). Furthermore, the students expressed confidence in its future application, and all agreed that this system is necessary and useful for biological experiments (100% agree or strongly agree; see Figure 4 Q5-Q6).

### Comparison of Students’ Views on the Laboratory Lectures Between the Control Group and the DMIS Group

In addition to evaluating the function of this system, its impact on laboratory lectures was also assessed through the questionnaire. A total of 36 out of 39 (92.3%) students completed the survey, including 20 from the DMIS group (100%, 20/20) and 16 from the control group (84.2%, 16/19). Given its Cronbach α of 0.98, the questionnaire demonstrates high reliability. The results showed a statistically significant difference in two of the four areas addressed in the survey (see Table 5). The first area is course content. Compared to the control group, the DMIS group more strongly supported the appropriateness of the course difficulty, the reasonableness of the schedule, and found it both enjoyable and challenging (*P*<.05). Similarly, students in the DMIS group reported higher self-efficacy and greater confidence in facing difficulties and challenges after finishing the lectures (*P*<.05). Notably, there was a statistical difference in overall satisfaction between the two groups, with the DMIS group scoring higher (mean 4.63, SD 0.50 vs mean 4.95, SD 0.22; *P*<.05). In the open-ended question, when discussing their biggest gains, 30% (6/20) of the DMIS group mentioned microscope technology, compared to 12.5% (2/16) in the control group. From the results, the implementation of this system has significantly improved the effectiveness of microscopy teaching. Furthermore, students have started to recognize the importance and application value of the microscopy technology.

**Table 5:** Students’ perceptions regarding the laboratory lectures.

Domains	Control group (n=16), mean (SD)	DMIS^a^ group (n=20), mean (SD)	*P* value
Course content	18.25 (1.88)	19.6 (1.00)	.02^b^
Teaching quality	18.75 (1.92)	19.75 (1.12)	.07
Self-efficiency	18.56 (1.93)	19.8 (0.89)	.03^b^
Teaching effectiveness	14.06 (1.44)	14.85 (0.67)	.07

^a^DMIS: digital microscopy interactive system.

^b^*P*≤.05 was considered statistically significant.

## Discussion

### Principal Findings

The core competency in microscopy lies in the precise manipulation of physical components (and potentially software interfaces) to optimize optical system parameters. This process, integrated with internalized knowledge of sample microstructure, enables accurate localization, clear identification, and effective visualization of target structures within the field of view, thereby yielding reliable scientific data or diagnostic evidence. Consequently, it yields reliable scientific data or diagnostic evidence. Therefore, the core objectives of microscopy education lie in knowledge internalization and mastery of operational skills—goals that have remained a persistent challenge for traditional teaching methods. This study implements a 5G-enabled digital interactive microscopy system in biological laboratory instruction, evaluating its effectiveness through students’ academic performance, learning experiences, and user evaluations of system functionality.

Our research findings indicate that, regarding academic performance, the knowledge test scores of the DMIS group were generally higher than those of the control group, with a significant difference observed in lecture 2. This disparity likely stems from the greater challenge associated with the microscope operation for counting unstained viable cells covered in that session. The high transparency of unstained viable cells renders them difficult to distinguish from the background, demanding a more robust comprehension of knowledge for identification and proficient operational skills for precise localization. This complex situation notably enhanced the value of the DMIS’s technical assistance. Real-time image sharing facilitated group discussion to reach a consensus on accurate identification. Furthermore, the laboratory report scores for the DMIS group were significantly higher than those of the control group, indicating that the DMIS group obtained higher quality microscopic images. This suggests that they effectively chose the objective lens for magnification, captured the images at the correct focal plane, and adjusted the light for the brightness of the images. In addition, they correctly found the cells of interest.

Actually, the microscope is a complex tool that requires not only the users’ skilled operation but also a solid understanding of morphological concepts for effective image analysis [7]. In the absence of immediate instructor feedback, students are prone to developing persistent misconceptions, often remaining unaware of these errors. More critically, such misunderstandings may lead to systematic misinterpretations, resulting in erroneous interpretations of the information conveyed by images. The real-time imaging capability of the DMIS enhances classroom interactivity, a factor consistently demonstrated by multiple studies [18,19] to improve student learning outcomes. Furthermore, this system retains the tactile feedback and direct manipulation experience that students value, which is missing in whole-slide imaging-based microscopy system [20,21]. These elements contribute to effective learning of microscopy skills and a deeper understanding of image analysis. The observed improved academic performance of the DMIS group supports this conclusion.

In the questionnaire survey, students in the DMIS group provided overwhelmingly positive feedback about the digital microscope interactive system. They generally agreed that it made capturing images clear, facilitated sample observation and picture editing, and supported group discussions. Only a single student reported issues due to his mobile device being incompatible with the app, which negatively impacted his experience. Similar compatibility issues can occur with applications [22] and warrant further optimization. However, this shortcoming did not diminish students’ confidence in the system’s future application, reflecting their recognition of its benefits. Notably, the DMIS group expressed higher satisfaction with laboratory lectures compared to the control group, especially regarding course content and self-efficacy. This may be attributed to their ability to independently navigate the challenges of microscope technology, enhancing their skills and confidence. As a result, students in the DMIS group identified that the microscope technique was what they gained the most from the class. Given the well-known positive impact of self-efficacy on learning [23], this experience will undoubtedly enable medical students to perform better in high-intensity medical training in the future.

For medical students, proficiency in microscopy is not merely a laboratory technique but a critical component in developing core professional competencies that profoundly influence future clinical practice. First, microscopy skills enhance morphological identification capabilities, serving as a prerequisite for accurate clinical diagnosis. Second, the process of adjusting microscope parameters helps establish efficient and precise visuospatial-motor coordination, thereby improving spatial positioning accuracy in future surgical procedures. In addition, it should be noted that microscopy remains fundamental to experimental research—an essential skill for physicians in China during their clinical practice.

For instructors, this digital microscope interactive system makes the organization of classroom teaching more convenient and efficient. Previously, due to limited laboratory funding, each room was only equipped with one microphotography device, which inevitably led to insufficient usage time for each student group. Now, students can take pictures with at least one available device at any time, greatly reducing the waiting time for equipment. Digital technology can achieve real-time monitoring and feedback, providing students with a personalized learning experience [1,24]. The convenience of 5G networks lies in their ability to transmit multimedia files without delay, enabling rapid feedback between teachers and students. This immediate communication helps resolve issues promptly and significantly enhances the quality of teaching. In addition, the interactive functions provided by the software’s main interface are valuable for personalized learning. For example, students can record operation videos using the function of macro image for postclass review and results analysis. If they have questions, these videos can be sent to the instructor for further improvement suggestions. More than that, the demonstration function offers 2 modes: students can freely view the synchronized transmission demonstrated by the teacher, while others can continue to use the system without disruption. While this study primarily centers on investigating how real-time operational visualization enhances microscopy technique acquisition through immediate feedback mechanisms, the system’s inherent classroom interaction features—such as video recording—prove instrumental in tracking learning progression and enabling formative assessment. These capabilities offer enhanced potential for developing precision-oriented pedagogical approaches in future microscopy instruction.

### Limitations

In this study, although it was observed that the digital microscope interaction system can enhance students' academic performance and learning experience, the sample size might be insufficient. In order to study the impact of this system more specifically, we only investigated the biology course where microscopy is most commonly used. This can lead to some application problems and differential performance that may arise in a large student population being overlooked. In addition, when the number of users increases, whether the system can maintain the stability of the current real-time transmission needs further analysis.

### Conclusions

The digital microscopy interaction system presented in this study represents a powerful tool for laboratory instruction. By implementing digital transformation and information technology enhancements to traditional optical microscopy, the system enables real-time visualization of student operations and facilitates immediate instructor feedback. These capabilities provide effective support for achieving the pedagogical objectives of microscopy skill development in medical students. Significant improvements in learning outcomes are evidenced by enhanced academic performance and superior learning experiences. Notably, students exhibited high satisfaction with the system and demonstrated markedly increased engagement during instructional sessions. As a teaching tool, the system’s rich interactive functionalities not only assist instructors in organizing teaching activities more efficiently but also support formative assessment of the learning process. This thereby creates favorable conditions for implementing personalized teaching models in the future. Collectively, these findings demonstrate the system’s considerable potential as a highly promising instructional aid within medical education.

## Abbreviations

DMIS: digital microscopy interactive system
LAN: local area network

## References

[1] Tokuç B, Varol G (2023). Medical education in the era of advancing technology. Balkan Med J.

[2] Maity S, Nauhria S, Nayak N, Nauhria S, Coffin T, Wray J, Haerianardakani S, Sah R, Spruce A, Jeong Y, Maj MC, Sharma A, Okpara N, Ike CJ, Nath R, Nelson J, Parwani AV (2023). Virtual versus light microscopy usage among students: a systematic review and meta-analytic evidence in medical education. Diagnostics (Basel).

[3] Yang Y, Cheng G, Xing X, Li Z, Zhang W (2022). Application of a multimedia-supported manikin system for preclinical dental training. BMC Med Educ.

[4] Valverde I, Gomez G, Byrne N, Anwar S, Silva Cerpa MA, Martin Talavera M, Pushparajah K, Velasco Forte MN (2022). Criss-cross heart three-dimensional printed models in medical education: A multicenter study on their value as a supporting tool to conventional imaging. Anat Sci Educ.

[5] Hortsch M, Girão-Carmona VCC, de Melo Leite ACR, Nikas I, Koney N, Yohannan D, Oommen AM, Li Y, Meyer AJ, Chapman J (2023). Teaching cellular architecture: the global status of histology education. Adv Exp Med Biol.

[6] Evans SJM, Moore AR, Olver CS, Avery PR, West AB (2020). Virtual microscopy is more effective than conventional microscopy for teaching cytology to veterinary students: a randomized controlled trial. J Vet Med Educ.

[7] Imreh G, Hu J, Le Guyader S (2024). Improving light microscopy training routines with evidence-based education. J Microsc.

[8] Yang J (2023). Technology-enhanced preclinical medical education (anatomy, histology and occasionally, biochemistry): a practical guide. Adv Exp Med Biol.

[9] Li Z, Zuo T, Wei X, Ding N (2023). ICT Self-efficacy scale: the correlations with the age of first access to the internet, the age at first ownership of a personal computer (PC), and a smartphone. Med Educ Online.

[10] Barrios-Ulloa A, Cama-Pinto D, Arrabal-Campos FM, Martínez-Lao JA, Monsalvo-Amaris J, Hernández-López A, Cama-Pinto A (2023). Overview of mobile communications in Colombia and introduction to 5G. Sensors (Basel).

[11] Kim M, Son MH, Moon S, Cha WC, Jo IJ, Yoon H (2025). A mixed reality-based telesupervised ultrasound education platform on 5G network compared to direct supervision: prospective randomized pilot trial. JMIR Serious Games.

[12] Xiaofeng Q, Hui C, Jin P, Yujie M, Yuan G (2024). Application of wireless intelligent microscopy interactive system in pathogenic biology experimental teaching [Article in Chinese]. Tongfang Knowledge Network (Beijing) Technology Co, Ltd.

[13] Hoyt G, Bakshi CS, Basu P (2025). Integration of an audiovisual learning resource in a podiatric medical infectious disease course: multiple cohort pilot study. JMIR Med Educ.

[14] Herman P, M Kibusi S, C Millanzi W (2025). Effectiveness of an interactive web-based clinical practice monitoring system on enhancing motivation in clinical learning among undergraduate nursing students: longitudinal quasi-experimental study in Tanzania. JMIR Med Educ.

[15] Brassil CE, Couch BA (2019). Multiple-true-false questions reveal more thoroughly the complexity of student thinking than multiple-choice questions: a Bayesian item response model comparison. IJ STEM Ed.

[16] Hubbard JK, Potts MA, Couch BA (2017). How question types reveal student thinking: an experimental comparison of multiple-true-false and free-response formats. CBE Life Sci Educ.

[17] (2023). Notice on Issuing the Measures for Ethical Review of Life Science and Medical Research Involving Human Subjects (National Health and Family Planning Commission Science and Education Document No. 4 [2023]). Central People's Government of the People's Republic of China.

[18] Liu K, Zhang W, Li W, Wang T, Zheng Y (2023). Effectiveness of virtual reality in nursing education: a systematic review and meta-analysis. BMC Med Educ.

[19] Ba H, Zhang L, Yi Z (2024). Enhancing clinical skills in pediatric trainees: a comparative study of ChatGPT-assisted and traditional teaching methods. BMC Med Educ.

[20] Ishak A, AlRawashdeh MM, Meletiou-Mavrotheris M, Nikas IP (2022). Virtual pathology education in medical schools worldwide during the covid-19 pandemic: advantages, challenges faced, and perspectives. Diagnostics (Basel).

[21] Başer A, Büyük Başak (2024). Bridging the gap in medical education: comparing analysis of light microscopy and virtual microscopy in histology. PeerJ.

[22] Iwata Y, Iwata Y, Iida H, Inamori M, Maeda S (2023). Using a smartphone application as a tool for english learning among medical staff and students in Japan. Adv Med Educ Pract.

[23] Hayat AA, Shateri K, Amini M, Shokrpour N (2020). Relationships between academic self-efficacy, learning-related emotions, and metacognitive learning strategies with academic performance in medical students: a structural equation model. BMC Med Educ.

[24] Ma H, Niu A, Tan J, Wang J, Luo Y (2024). Nursing students' perception of digital technology in clinical education among undergraduate programs: A qualitative systematic review. J Prof Nurs.

